# Recent progress and perspectives on the relationship between hyperuricemia and periodontitis

**DOI:** 10.3389/fimmu.2022.995582

**Published:** 2022-11-16

**Authors:** Wenxue Hou, Xiaomin Xia, Ying Li, Hanlin Lv, Jie Liu, Xue Li

**Affiliations:** ^1^ Department of Stomatology, The Affiliated Hospital of Qingdao University, Qingdao University, Qingdao, China; ^2^ School of Stomatology, Qingdao University, Qingdao, China; ^3^ Dental Digital Medicine & 3D Printing Engineering Laboratory of Qingdao, Qingdao, China; ^4^ Dental Biomaterials Technology Innovation Center of Qingdao, Qingdao, China

**Keywords:** periodontitis, hyperuricemia, uric acid, inflammation, toll-like receptors, monosodium urate

## Abstract

Periodontitis is one of the most prevalent diseases in oral cavity, which could not merely lead to the destruction of supporting or surrounding tooth structures but also affect the whole-body health such as the digestive and nervous systems. Epidemiological investigations suggested that in some developed countries, more than 45% or even 50% population were suffering from periodontitis. However, the prevalence increases with age remarkably and it is investigated that a high prevalence (>50%) is affecting the elderly who is over 65 years old. There is an increasing interest in the direct and indirect relationships between periodontitis and hyperuricemia. Currently, hyperuricemia has become the second major metabolic disease in modern society and the prevalence of hyperuricemia among adult males and females was 21.7% and 14.4% respectively. As an inflammatory disease associated with various systemic diseases, periodontitis may have certain connections with hyperuricemia. Partial existing research announced that hyperuricemia could act as an inhibitory factor for periodontitis, while other scholars presented that a high uric acid (UA) level was more likely to aggravate inflammatory immune response and lead to more serious tissue destruction. This article provides a detailed and comprehensive overview of the relationship underlying hyperuricemia and periodontitis in the molecular mechanisms. Given the impact of hyperuricemia, this review could provide insight into its comorbidities.

## Introduction

Periodontitis is a chronic multifactorial inflammatory disease caused by pathogenic biofilm and results in progressive destruction of the tooth-supporting apparatus including alveolar bone resorption, which could lead to tooth loss (1). Although it is manifested clinically by swollen, bleeding gums, weak chewing, and deep periodontal pocket, the onset of periodontitis is usually occult. Furthermore, periodontitis could attack the susceptible at any age, and severe periodontitis affected approximately 10% of the world population (2). In China, the prevalence of periodontal disease among adults is as high as 80% or even 90% (3, 4). And the prevalence of periodontal disease can be as high as 42.2% among people aged 30-79 years in the United States, of which 7.8% suffer from severe periodontitis (5). The American Dental Association (ADA) announced that ‘Oral health is a functional, structural, aesthetic, physiologic and psychological state of well-being and is essential to an individual’s general health and quality of life’ (6). Therefore, it is widely recognized that periodontitis is closely related to a person’s physical and mental health care. Plenty of research has presented that several systemic conditions like diabetes mellitus, cardiovascular diseases, and Alzheimer’s disease were intimately associated with periodontitis (7–10). Hence, periodontitis is not merely an ailment that impresses oral health but also has an indirect influence on systemic diseases.

Hyperuricemia is a common disorder impacting patients of all ages and genders that could lead to the formation of tophus in the peripheral joint which is clinically manifesting as gouty arthritis. At present, approximately 120 million people in China and 38 million individuals in the United States are suffering from such torturing disorders (11). In addition, with human nutrition intake further refined, the prevalence rate is increasing swiftly and has become a serious global challenge in public health. Hyperuricemia is a metabolic disease caused by abnormal purine metabolism and is mainly manifested by increased uric acid (UA) levels in the blood. In addition, hyperuricemia could also cause monosodium urate (MSU) crystal deposition in tissues, which may promote chronic inflammation (12). The deposition of MSU crystals in joints or viscera and the subsequent formation of tophus may make hyperuricemia deteriorate continuously into gout when the overall condition gets much more severe (13). As a common systemic disease, epidemiological investigation and experimental studies have queried the potential correlation between periodontitis and hyperuricemia (7, 14). Surrounding the mutual relation, two prevailing controversies have been presented: partial studies have shown some positive effects that high levels of soluble UA may be a protective factor for periodontitis; while others insisted soluble UA could trigger the onset of sterile immune-inflammatory responses and then exacerbate the non-reversible damage of supportive tissues *via* crossing inflammatory signaling pathway.

**Figure 1:**
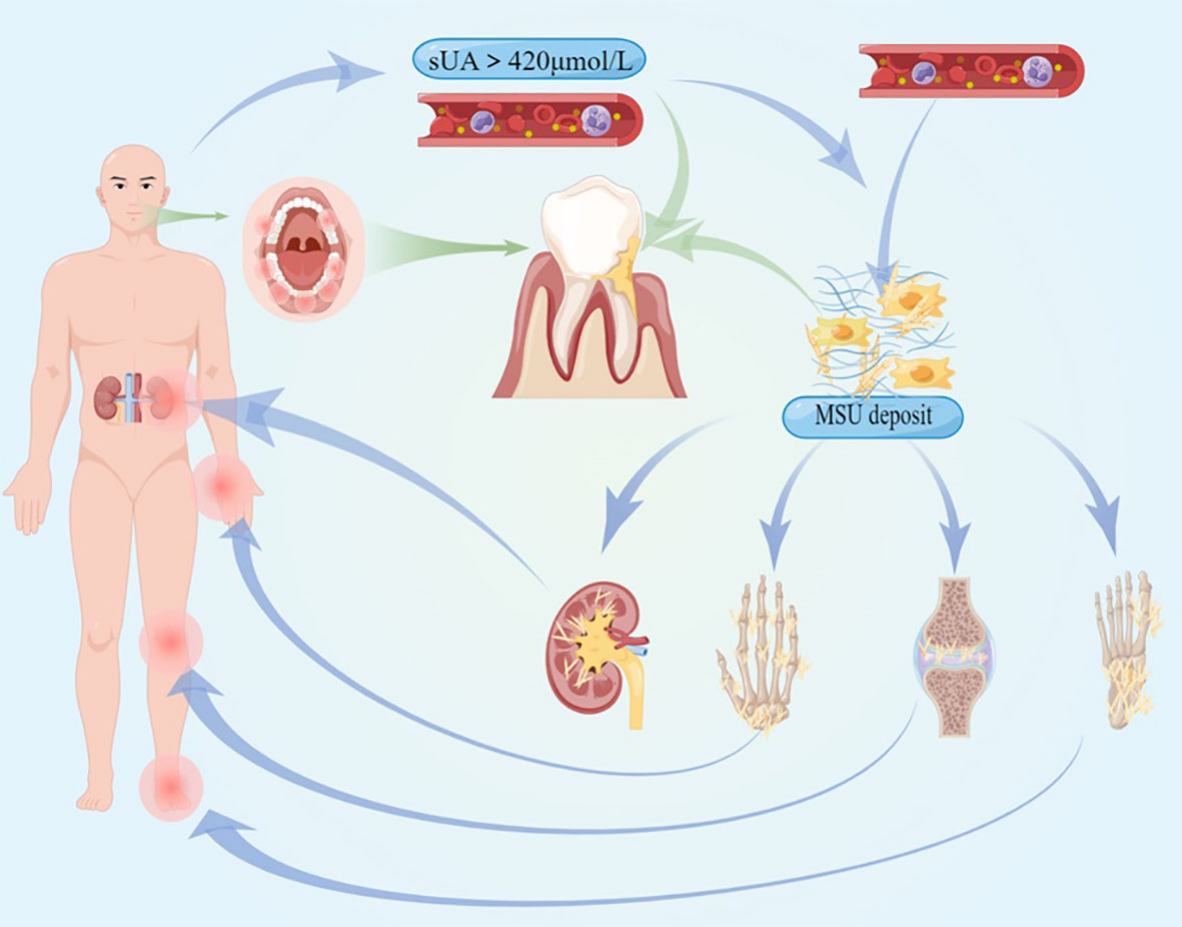
The pathogenesis and clinical symptoms of hyperuricemia and gout are illustrated, and the systemic inflammation they cause could affect the procession of periodontitis.

However, it has not been as widely discussed in the medical literature about the influence of hyperuricemia in periodontitis as diabetes mellitus. Thus, this review will discuss the concrete evidence of fundamental experiments and epidemiological surveys to explain the possible comorbidity between periodontitis and hyperuricemia. The inadequate parameters will be dissected as well. This review covered the pathogenic mechanism of periodontitis, the systemic effect of elevating soluble UA levels, and the facilitation of hyperuricemia in periodontal immune-inflammatory response (the interaction relationship between hyperuricemia and periodontitis). Among the above, the former two parts consist of the foundation of the last but most significant part. Meanwhile, this review will also hint to physicians and periodontists about the potential effect and therapy based on the premise of their interaction (refer to **Figure 1**).

## Pathogenic mechanism of periodontitis

### Dental plaque— the initiation factors

Homeostatic biofilm usually predominates in the healthy oral cavity, restraining the growth and reproduction of pathogenic microorganisms. However, when pathogenic microorganisms reproduce promptly and govern the activity of the whole plaque biofilm, dysbacteriosis could occur and then become sufficient for various oral diseases, such as caries and periodontitis (15). Periodontitis is a chronic and inflammatory oral disease initiated by dental plaque located around the gingival sulcus. By using direct PCR, Tanner et al. discovered that *Tannerella forsythia* and *Porphyromonas gingivalis* (*P. gingivalis*) were linked to early periodontitis (16). Socransky et al. firstly proposed a diagrammatic representation of the periodontal pathogen in the light of the association strength with periodontal lesions, which showed a highly-relevant red complex, including *P.gingivalis, Treponema denticola*, and the relevant orange complex, including *Prevotella intermedia*, *Campylobacter rectus* and so on (17). The relevant pathogens included Gram-negative and Gram-positive members (18). By contraries, Herrero et al. presented that it seemed like the synergy between the host immune-inflammatory response and the dysbiotic biofilms but not the dysbiotic biofilm itself that lead to periodontal lesions (19). More exactly, Lamont et al. recognized inflammation and dysbiosis interplayed in a reciprocally reinforced manner, eventually initiating or aggravating periodontitis (20).

### Pathogenic mechanism of periodontitis in the state of hyperuricemia

**Figure 2:**
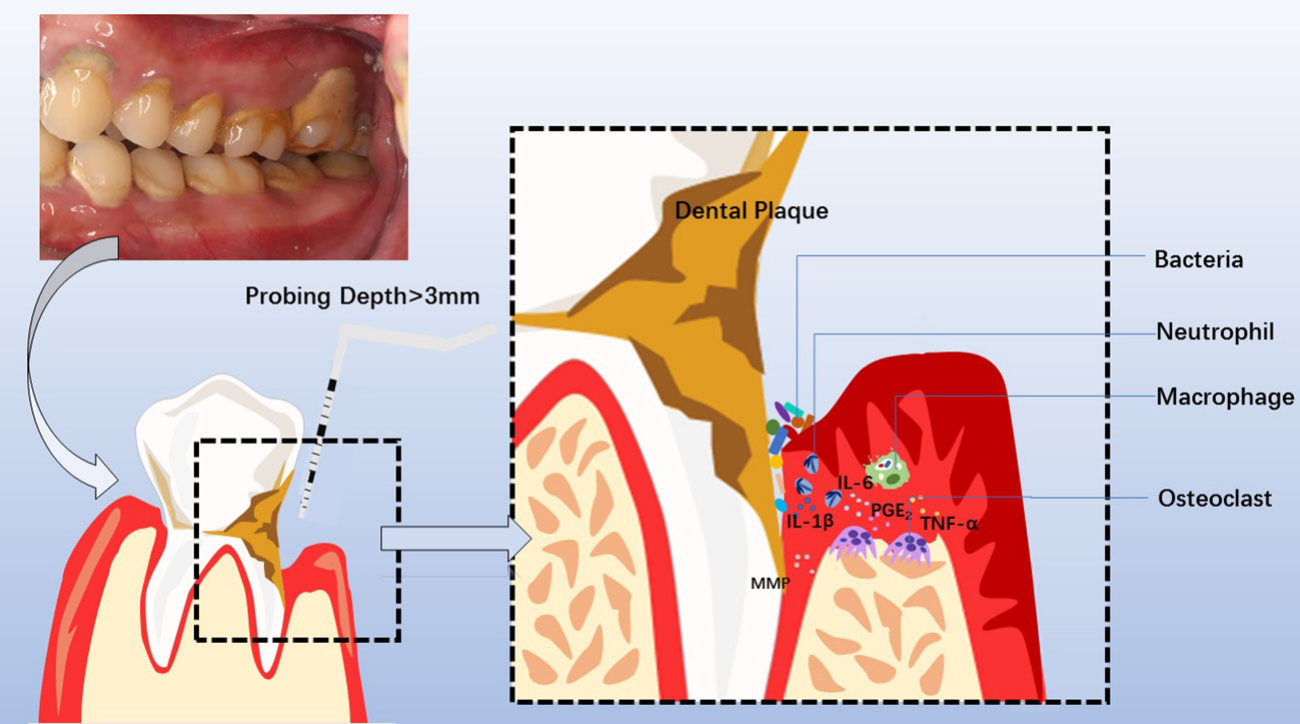
Clinical manifestations, diagnosis, pathogenesis of chronic periodontitis, and involved cytokines and cells. Periodontitis is manifested clinically by swollen, bleeding gums, breath malodor, and deep periodontal pocket. At the diseased sites, the probing depth of the deep periodontal pocket is more than 3mm, with bleeding on probing or periodontal pyorrhea. Dental Calculus and dysbiotic dental plaque at the cervical tooth extend to the bottom of the gingival sulcus or periodontal pocket, facilitating pathogens to invade the sulcular epithelium and junctional epithelium. Subsequently, neutrophils and macrophages are recruited to the diseased sites, releasing MMP, PGE2, IL-1β, IL-6, TNF-α, et al. The overactivated inflammatory response causes periodontal tissue destruction, including collagen degradation and resorption of the supporting alveolar bone mediated by osteoclast. Consequently, the development of a deep periodontal pocket increases tooth mobility until loss.

Periodontitis involves periodontal even general immune-inflammatory responses triggered by plaque microorganisms or only by their products (15, 21–23). Séguier et al. verified a rapidly rising of macrophages or monocytes in diseased gingival connective tissue compared with those in healthy tissue (24, 25). A much higher level of matrix metalloprotease (MMPs) is also caused by the aggregation of macrophages or monocytes, which could bring out serious collagen breakdown in periodontitis (25). In periodontal ligament fibroblast, elevated inflammatory cytokines such as IL-1β, IL-6, and tumor necrosis factor-α (TNF-α) could be observed when exposed to dysbiotic biofilms or LPS (24, 26). And prostaglandin E2 (PGE2) could promote the pro-inflammatory M1-like macrophage polarization, which refers to the killer-polarization state in which macrophages exert the cell cytotoxic function and IL-8 could recruit the neutrophils (27). These physiological changes could augment inflammatory responses collectively. More importantly, there are numerous inflammatory signal pathways involved including nuclear factor (NF)-κB, NLRP3, or MAPK. The interwoven signaling pathways will not only intensify the inflammatory reaction but also strengthen the interaction between systemic diseases and periodontitis (refer to **Figure 2**).

#### TLRs in innate and acquired immune responses

During the process of inflammation onset, the recognition of pathogen-associated molecular patterns (PAMPs) by the pattern recognition receptors (PRRs) such as Toll-like receptors (TLRs) is critical for the host-microbe interaction, as well as the subsequent innate and adaptive immune responses (15). Usually, the components of the bacterial cell wall are common PAMPs, such as lipopolysaccharide (LPS) liberated from the Gram-negative bacteria, peptidoglycan, and lipoteichoic acids (LTA) that form part of Gram-positive bacteria cell wall or cell wall lipoproteins (28). Antigen-presenting cells identify the PAMPs *via* the TLRs family and then activate the intracellular signaling pathway to upregulate the expression of inflammatory cytokines, chemokines, and interferons.

The stimulation to TLRs from PAMPs could recruit adaptors including MyD88, TIRAP, TRAM, and TRIF (29). In MyD88 independent pathway, the last two adaptors promote type-I IFN and IFN-inducible genes *via* the phosphorylation of interferon (IFN)-regulatory factor (IRF3) during the late phase of NF-κB activation (30). While the NF-κB in the MyD88-dependent pathway during the early phase or MyD88-independent/TRIF-dependent pathways is mainly activated to produce proinflammatory cytokines, such as IL-1β (31). The synergistic activation of both the MyD88-dependent and MyD88-independent/TRIF-dependent pathways might explain the violent inflammatory response like endotoxic shock (30). Namely, these pathways are principally involved in TLR-mediated expressions of genes that encode inflammatory cytokines (29, 32). The MyD88-dependent pathway involves the early phase of NF-κB activation, which leads to the production of pro-inflammatory cytokines (30), aggravating the damage extent and scope of periodontal inflammation.

In addition, the following observations suggested the close relationship between TLRs and adaptive immunity or innate immunity. Abundant evidence supported the point that TLRs could recognize self- and non-self-antigens accurately as well as promote the phagocytosis of antigens and maturation of dendritic cells (DC) in innate immunity (33). Mature DC, the most powerful antigen-presenting cell, is the key to the activation of T and B cells in antigen-specific adaptive immune responses (33). T and B cells have been found to express various TLRs (such as TLR 1, 2, 4, and 9), whilst they are capable of responding to TLR ligands like LPS (34, 35).

#### LPS/MDP-NLRP3-caspases

The activation of the NLRP3 inflammasome by LPS can be achieved by either canonical or non-canonical NLRP3 inflammasome pathways. NLRP3 is expressed in both human and mouse macrophages, dendritic cells, neutrophils and epithelial cells (36). Extracellular LPS could activate NF-κB *via* TLR4 in the canonical pathway and promote the transcription of NLRP3 and pro-IL-1β, then resulting in the assembly of NLRP3 inflammasome with K+ efflux and ATP (36). NLRP3 inflammasomes could promote pro-caspase-1 to cleave itself and then form active caspase-1 in both human and mice. The active caspase-1 could act on gasdermin D to induce cell lysis and release activated IL-1 and IL-18 to trigger inflammatory response (36, 37). While in the non-canonical pathway, LPS gets internalized into the cytosol of human monocyte independent of TLR4 (37). Stimulation from LPS could lead to the activation of caspase-11 in mouse and caspase-4/5 in human, and then inducing pyroptosis of gasdermin D which results in K+ efflux with the release of ATP in both pathways. While the latter one is specifically targeted to pathogens that invade the cytoplasm (38, 39). In the two pathways described above, auto-inhibited NLRP3 gets activated and forms an active NLRP3 inflammasome that drives the active caspase-1 to process pro-IL-1β into IL-1β. Caspase-1 can reversely act on gasdermin D to accelerate K+ efflux in canonical and non-canonical pathways, facilitating the release of IL-1β cyclically (40).

In addition to the inflammatory response of periodontal tissue *via* TLRs, Martinon et al. discovered that muramyl dipeptide (MDP), the degradation product of bacterial peptidoglycans, could trigger the assembly of the NLRP3 inflammasome complex (41). NLRP3 is the essential component of the human NLR family (42). NLRP3-mediated active caspase-1 could cleave proIL-1β into mature IL-1β (41). Together with other inflammatory cytokines, the increasing IL-1β secretion may augment the damage of targeting tissue (43). In addition, caspase-dependent IL-18, a proinflammatory cytokine promotes autoimmune responses to specific antigens in T cells (44). IL-18, which involves in MSU-induced sterile inflammation, can exaggerate inflammatory responses in periodontitis when released by Natural Killer-Like B cells (45). Martinon et al. proposed that we could regard the activation of NLRPs as an upstream step in the IL-1R and IL-18R signaling cascades due to their capacity to recruit MyD88 involving NF-κB and thus directly linked intracellular pathogen recognition to signaling pathways shared with TLRs (42). What’s more, NLRP1 and NLRP2, components of the NLRP family, could also sense the presence of MDP (43).

#### MAPK signaling pathway

Huang et al. demonstrated that LPS could also induce the production of IL-6, TNF-α, and phosphorylated janus kinase (JAK). This increasing pro-inflammatory mediator expression is associated with the phosphorylation of MAPKs within 15–30 min after LPS stimulation (46–48). MAPK signaling pathway leads to the release of TNF-α and IL-6. The latter could bind to the transmembrane IL-6 receptor and subsequently activate JAK, followed by the phosphorylation of the signal transducer and activator of transcription (STAT) 1/3. STAT1/3 could bind to the specific target DNA and trigger the inflammatory cytokines of TNF-α, IL-2, and IL-6 eventually (46). Besides, in the NF-κB signaling pathway, the phosphorylation of MAPK could lead AP-1 together with JNK and p38 to translocate into the nucleus and result in the upregulation of the proinflammatory cytokines (29, 30).

Damage-associated molecular pattern (DAMP), such as UA, is produced or released by damaged or dying cells. Although momentous for tissue repair and regeneration, DAMPs could bring about sterile inflammation (49). In sterile conditions such as ischemic injuries, trauma, tumors, tissue transplants, and autoimmune diseases, robust immune responses could be elicited by DAMPs (50, 51).

UA usually exists in the ionic state in body fluids, but in patients with hyperuricemia or gout, it can precipitate into a large number of crystal deposits, and the majority of crystal components are monosodium urate (MSU). Monosodium urate crystals have a specific ability to stimulate DCs maturation and promote the priming of T cells *via* TLR2/4 compared with other crystals like allopurinol crystals and basic calcium phosphate. Whereas soluble UA does not act as stated above (52, 53), Lu et al. detected urate crystals in the kidney interstices of mouse models with hyperuricemia under polarized light (54). Wang et al. discovered the subclinical MSU crystal deposits in 15% of patients with asymptomatic hyperuricemia by foot/ankle dual-energy CT scans (55). Therefore, it is deduced that urate crystals also exist in hyperuricemia patients. These crystals may play a certain role in the development of periodontitis.

#### Soluble UA/MSU crystal-TLRs

Soluble urate could specifically downregulate the IL-1Ra (IL-1 receptor antagonist) secretion even at a lower dose and enhance the IL-1β production dose-dependently when TLR ligands are presented (56). However, it is unable to induce IL-1β secretion on its own when MSU crystallization is absent microscopically (56, 57). Cabău et al. put forward that the abnormal level of soluble UA in body fluid could augment inflammatory effect through resetting epigenetic reprogramming and immunometabolism of innate immune cells, which was termed as trained immunity (57–59). The above process is independent of the adaptive immune system. Experiments revealed that MSU crystal could induce the production and release of active IL-1β under the premise of TLRs synergizing (60–62). Moreover, Martinon et al. demonstrated that MSU crystals could promote the maturation of IL-1β and IL-18 in THP1 cells and purified human monocytes with the presence of caspase-1 (60). While in MyD88 deficient peritoneal macrophages, which is the key element of the NF-κB signaling pathway, MSU could still activate caspase-1 and lead to the process of IL-1β (29). The above discovery implies that this activation by MSU crystals could also be TLR-independent and consistent with the effect of the relevant inflammasome. From the deductions based on these experiments, we could conclude that MSU crystals could induce the expression of inflammatory IL-1β as DAMP through TLRs partially depending on NF-κB activation.

#### Soluble UA-Pras40/PI3K-Akt-mTOR

The rising level of phosphorylated proline-rich AKT substrate 40kD (PRAS40) in monocytes was revealed when stimulated by soluble urate. Phosphorylated PRAS40 could strongly activate mTOR which is also known as the PRAS40-Akt-mTOR signal pathway (63). The above two physiological changes both inhibit autophagy (14, 57, 63). Besides, Ganeshan et al. illustrated that soluble UA could induce the differentiation of T cells through the PI3K-Akt-mTOR pathway, which regulates immune metabolism in innate immunity (14, 64) and connects acquired immunity to an intensification of immune-inflammatory responses. According to the research from Pare et al., MSU could stimulate p38MAPK phosphorylation and induce MAPK activation *via* the p38-PI3K-Akt signaling pathway and thereby increasing the neutrophil cytokine synthesis (65). Crisan et al. indicated that cells exposed to high levels of soluble UA could exhibit lower autophagy activity and less production of ROS than their counterparts (14). Although the decline of ROS implied the antioxidation activity of soluble UA, the ultimate effect was still the onset of sterile inflammation. This review noted above maintained that 1) soluble UA could depress autophagy *via* Akt-PRAS40 and lead to the inactivation failure of inflammatory cytokines which play a pivotal role in inflammation persistence; 2) soluble UA could facilitate immune responses *via* the PI3K-Akt-mTOR signaling pathway.

#### Soluble UA/MSU-NLRP3-(IL-1β)

The researchers also concluded that MSU crystals could boost inflammation by their capacity to initiate and accelerate the assembly of NLRP3 inflammasome (60). In peritoneal macrophages of murine, MSU-induced IL-1β activation could be blocked completely by caspase-1 inhibitor and ASC deficiency but incompletely by MyD88 deficiency (60). ASC is a crucial adaptor for the recruitment of caspase-1 to the NLRP inflammasome platform. It could be further confirmed that it is the MSU crystals that cause the release of IL-1β by activating the NLRP3 inflammasome, which is consistent with our previous findings in human monocytes. MSU crystals injected in wild-type C57BL/6 mice trigger a significant increase in the recruitment of neutrophils (60). Neutrophils, macrophages, and monocytes are essential participants in acute and chronic periodontal inflammation. NLRP3 inflammasome could start the downstream inflammatory responses by facilitating IL-1β synthesis and release (66, 67). MSU could also activate caspase-1, which is critical for the maturation of inflammatory IL-1β and IL-18 (68, 69). Prior studies have detected the increased IL-6 when challenged with an intravenous UA solution in healthy volunteers and this variation cannot be reversed by rasburicase which is an effective UA-lowering drug (70). Further analysis showed that lowering UA does not have any effect on inflammation resolution, hinting that soluble UA is likely to bring about the exacerbation and prolonged process of inflammation.

## Systemic effects of hyperuricemia

### The antioxidant effects of UA

UA excretion usually depends on the kidneys, and the reabsorbing part accounts for 90% of the gross body output (71). The evolutionary preserving mechanism implies the positive aspect of urate, such as the antioxidant capacity (72, 73). El et al. discovered that soluble UA might be the main component in plasma that played an antioxidant role (74). Soluble UA could protect native low-density lipoprotein (LDL) against Cu2+-induced oxidation and increase the oxidation of already oxidized LDLs (75). It’s worth noting that the manifested antioxidative characteristics of soluble UA presumably depend on the chemical microenvironment it resides.

Out of anticipated, some scholars verified that soluble UA could only play an antioxidative role in neurodegenerative diseases (76). Multiple clinical and epidemiological studies have revealed an intriguing phenomenon that low-level urate could exert a detrimental impact on neurodegenerative diseases, and higher but still normal urate levels may associate with neuroprotection against Parkinson’s disease. While the urate levels in the blood and cerebrospinal fluid have been found to associate with a lower rate of disease progression in multiple sclerosis, Huntington’s disease, Alzheimer’s disease, and amyotrophic lateral sclerosis (57, 77–80). Research by Zhang et al. suggested that urate could significantly enhance glutathione expression by activating Akt/GSK3β/Nrf2/GCLC pathway and scavenging ROS, thus offering neuroprotective effects on motor neurons against oxidative stress (80). Many scholars asserted that immune activation is the potential mechanism for urate-induced neuroprotection rather than its antioxidation property. In short, the antioxidant capacity of the urate or the immune changes it causes may influence the inflammatory progression.

### The impact of UA on bone metabolism

Veronese et al. concluded that hyperuricemia had a protective effect on bone metabolic disorder independently of physiological bone metabolism (81). Yan et al. recognized that soluble UA had a protective effect on bone metabolism by taking Chinese males and postmenopausal females in Shanghai as their research subjects (82). The estrogen levels in postmenopausal females decrease drastically, contributing to the significantly increased risk of osteoporosis. Consequently, soluble UA may be a protective factor for maintaining bone density. However, some researchers hold a different opinion. Lin et al. discovered the phenomenon that UA could increase bone fracture risk in hyperuricemia or gout arthritis patients due to the increased bone resorption and decreased bone formation which was resulting from oxidative stress in other uncertain mechanisms (83). Bone loss and resorption could also be further aggravated in soluble UA-induced osteoporosis by Vitamin D deficiency and secondary hyperparathyroidism (83).

## The possible interactive relationship between hyperuricemia and periodontitis and relative illustration

Based on these results, we made the following daring hypothesis-there may be more or less association between periodontitis and hyperuricemia. In fact, the interrelationship between periodontitis and other common systemic diseases has been verified in succession these years. Holm et al. discovered a higher incidence of undiagnosed diabetes in the patients who sought dental treatment, and higher HbA1c above the threshold for pre-diabetes could also be observed (84). Periodontitis was more common in patients with first acute myocardial infarction than it was in controls (85). Inversely, the incidence of acute myocardial infarction also increased significantly among periodontitis patients (85). As a typical systemic disease, although the elevated soluble UA levels and MSU crystals may not cause any specific symptom, hyperuricemia is prone to affect the local inflammation in intricate and interactional methods. The potential threat of this metabolic disease to the kidneys as well as the whole body’s bones and joints should not be underestimated. Hyperuricemia and gout will inevitably affect the local diseases, after the heart, brain and other organs dysfunction secondary to renal function injury. Periodontitis, on the other hand, is an established local disease associated with a variety of systemic diseases. Diverse conclusions or deductions were illustrated to dissect the possible outcomes and relevant interpretations based on theory, existing research, and available data.

### Conclusion of the aforementioned inflammatory signaling pathway cross-talk

**Figure 3:**
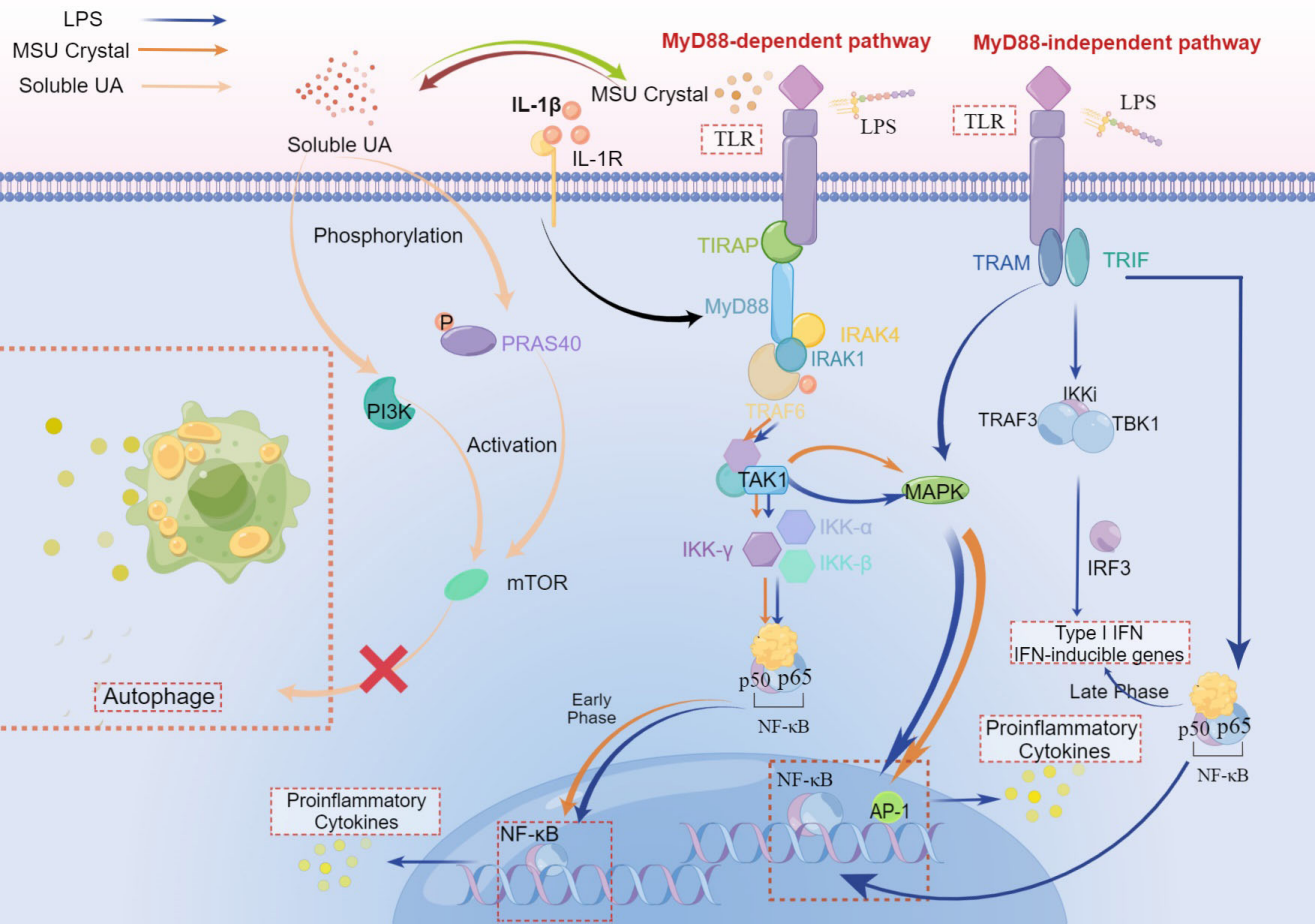
The crosstalk of LPS, MSU crystal, and soluble UA in immune-inflammatory signal pathway. Both MSU crystal and LPS could activate TLRs to recruit TIRAP and MyD88. The stimulation of MyD88 molecule by ligands primes the interaction of MyD88 and IRAK-4, which allows IRAK-1 and TRAF-6 to associate with MyD88 and subsequently recruits TRAF6, leading to the activation of TAK1 to stimulate MAPK that upregulates the inflammation responses *via* AP-1. Followed by activation of TAK1, the IKK complex (inhibitor of nuclear factor-κB (IκB)-kinase complex), which consists of IKK-α, IKK-β, and IKK-γ, translocates NF-κB to the nucleus, increasing the pro-inflammatory cytokines in the early phase, pro-resolution cytokinesis in the late phase. This is a MyD88-dependent pathway. In the MyD88-independent pathway, TLR is activated by LPS and then recruits TRAM and TRIF which induces the combination of TRAF3, TBK1, and IKKi to activate IRF-3, which plays a critical role during the late phase of NF-κB activation (30), aiming at Type I IFN and IFN inducible genes. In this pathway, the translocation of NF-κB can both elevate levels of pro-inflammatory cytokines and IFN. In addition, MAPK can also be activated after the recruitment of TRIF and other adaptors. IL-1β binding to IL-1R plays an accelerating role in the MyD88-dependent pathway. Soluble UA phosphorylates PRAS40, leading to the activation of mTOR, which can also be realized *via* PI3K. While both PRAS40 and mTOR inhibit autophagy, suppressing the inactivation of inflammatory cytokines.

**Figure 4:**
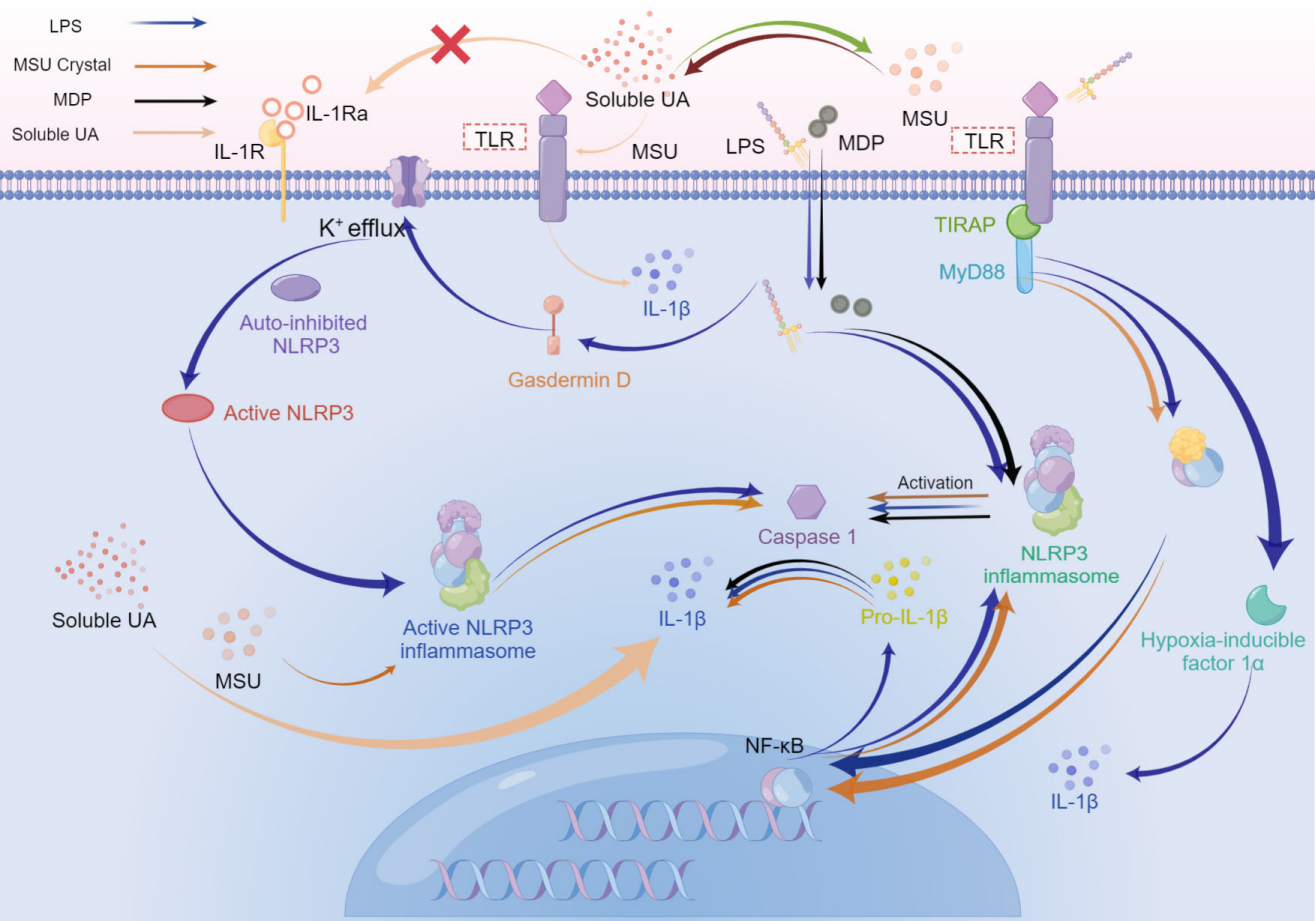
The possible superposition of bacterial inflammation in periodontitis and sterile inflammation in hyperuricemia or gout when the above diseases coexist. Activation of Toll-like receptors, especially Toll-like receptor 4, switches metabolism from oxidative phosphorylation to glycolysis in immune cells, which increases HIF-α, and enhances IL-1β production. During the late phase of the NF-κB pathway stimulated by LPS, the final effect is the upregulation of pro-resolution cytokines. LPS, MDP, and MSU initiate the assembly of NLRP3 inflammasome, activating caspase-1, which cleaves pro-IL-1β into mature IL-1β. While the production of NLRP3 is related to the MyD88-dependent pathway. Besides, IL-1β could be promoted by simultaneous stimulation from soluble UA and MSU crystals or MSU crystals solely. Soluble UA decreases the IL-1Ra, without assistance from other stimuli. LPS activates gasdermin D, resulting in K+ efflux. Subsequent assembly of NLRP3 inflammasome drives the activated caspase-1 cleave pro-IL-1β into IL-1β. Caspase-1, in turn, could stimulate gasdermin D to accelerate K+ efflux, accompanying the release of IL-1β.

As we could learn from the previous and present research, the signal pathway crossing between hyperuricemia and periodontitis included the following 4 points (refer to **Figure 3** and **Figure 4**):

1) As PAMPs, periodontal pathogens identify TLRs to stimulate the NF-κB signal pathway, while MSU crystals could also activate NF-κB as DAMP with the synergy of TLRs. Periodontal pathogens and MSU crystals initiate similar signaling pathways, which could enable the levels of inflammatory cytokines to get strikingly higher than when a single factor activates NF-κB.
2) On the whole, the NLRP3 inflammasome could be activated by endogenous DAMPs such as MSU and PAMPs like bacterial toxins. Derived from the stimulus described above, the activated NLRP3 inflammasome could stimulate caspase-1, which will process the pro-IL-1β and pro-IL-18 into mature IL-1β and IL-18 to induce pyroptotic cell death as inflammatory factors. Both MSU crystals and MDP could stimulate a variety of immune cells like macrophages, and initiate the assembly of NLRP3 inflammasome to accelerate the production and release of active IL-1β. The synergies may be gained with the presence of the two aforementioned stimuli virtually.
3) MSU could enable the phosphorylation of p38 and MAPK at a low level, which may amplify the effect of phosphorylation of MAPK stimulated by LPS to induce pro-inflammatory cytokines. Consequently, the collective effect could lead to a more violent inflammatory response.
4) Soluble UA could restrain autophagy through the Akt-PRAS40 signaling pathway, which results in the inhibition of inflammatory cytokines inactivation induced by periodontal pathogens. In addition, soluble UA could also cause the persistence of elevated levels of inflammatory cytokines either locally or systemically, which cannot be reversed by effective UA-lowering drugs at least for a short time.

### The immune enhancement effect of UA or MSU for periodontitis

Both salivary and plasma inflammatory IL-18 levels could rise significantly in the chronic periodontitis patients compared with the control individuals, which is consistent with the alteration in patients with high soluble UA levels (86). Crisan et al. found the elevated production of IL-1β and the diminished IL-1Ra in uric acid-primed monocytes (14, 56). Scholars also detected the inflammatory enhancement effect of MSU compared with the single effect of LPS (14). The superposed effect of LPS and MSU crystal could increase IL-1β and decrease IL-1Ra sharply compared with LPS restimulation after soluble UA priming (14).

Research related to the interaction between high soluble UA levels and periodontitis is still deficient and not profound relatively. While with the help of research about the interplay between high soluble UA levels and inflammation in other tissue, this review attempts to explore the mechanism of interplay between high soluble UA levels and periodontitis indirectly. Due to the capacity to increase the production of soluble UA, alum has been applied to medical practice as a vaccine adjuvant for a long time to enhance the immune response (87). Yan et al. concluded that soluble UA produced by injured cells was one of the endogenous adjuvants in cells (82). In mice treated with allopurinol and uricase which can reduce the soluble UA levels effectively, scholars observed a reduced number of primed antigen-specific cytotoxic T lymphocytes with lower plasma UA concentrations, compared with mice that are injected with larger numbers of injured cells (88), hinting the linchpin between inflammation and soluble UA. In the primary bone marrow-derived DC culture to which soluble UA was added, the expression of CD86 increases significantly, which leads to the initiation of T cell response (88).

Liu et al. demonstrated that the Type 1 CD8+ T cells were the main participants in the inflammatory cytotoxic responses when exposed to LPS (89). A significantly increased number of Type 1 CD8+ T cells and the ratio of Type 1 CD8+ T cells to Type 2 CD8+ T cells were detected (89). Simultaneously, more pro-inflammatory cytokines and further destructions in the tissue that indicated enhanced immune and inflammatory responses may induce lasting chronic inflammation (89). Of note, Chen et al. found the incidence rate of periodontitis in the subjects with gout was significantly higher than that in the comparison cohort, while the risk of periodontitis in gout patients using colchicine was significantly lower (90). Furthermore, colchicine is an ancient treatment for gout. The deficiency of this experiment is that not only hyperuricemia but also numerous distinctive systemic diseases including diabetes mellitus and cardiovascular disease are closely related to periodontitis, in which specific illnesses are regarded as potential covariates by researchers (9). Hence, it is inevitable to confuse which element promotes the progression of periodontitis directly or indirectly.

### The effect of periodontal pathogens on salivary or plasma UA levels and the hyperuricemia may promote the growth and reproduction of related periodontal pathogens

*P. gingivalis* and *Prevotella intermedia* are both closely related to the initiation and development of periodontitis, while *Serratia marcescens* is a conditional pathogen. In the *in vitro* experiments, applying *P. gingivalis* (MOI (multiplicity of infection) of 100) to stimulate THP-1 macrophages for 24h could lead to the significantly increased release of soluble UA (91). Rizal et al. found that the salivary UA levels of periodontitis patients were higher than that of gingivitis patients (92). Subsequently, the level of plasma UA in chronic periodontitis patients was found to be significantly increased compared with that in healthy individuals (86). In addition, some researchers reported that the increased serum UA may lower the pH in the oral cavity or periodontal pocket, which could promote the growth and the reproduction of acid-producing periodontopathogens like *Prevotella* and may increase the incidence of periodontitis (93). While with the presence of gingipain inhibitors which can reduce the activity of the gingipain enzyme, a significant reduction of soluble UA release at or near the control levels could be observed (91). The above experiments hint that the increase of soluble UA in periodontal tissues may be caused by gingipain produced by *P. gingivalis* and revealed the increased UA in saliva or blood of periodontitis patients. Indeed, there is also a contradiction that the optimum pH of proteolytic activity in *P. gingivalis* is pH 7.5 to 8.0, which is not consistent with an acidic environment caused by soluble UA (94).

However, Liu et al. found that compared with healthy controls, the number of *Prevotella intermedius* in the saliva of hyperuricemia patients or gout patients increased significantly, while *Serratia marcescens* was not detected in hyperuricemia or gout patients (95). Therefore, from these experiments, we could speculate that hyperuricemia and gout could promote the growth and reproduction of specific periodontal pathogens in saliva or gingival crevicular fluid and inhibit opportunistic pathogens. Consequently, the change in the oral microenvironment causes dysbiosis, which presumably causes periodontal disease. In turn, the rapid growth and reproduction of periodontal pathogens or the dysbacteriosis in adjacent or remote tissues and organs could also cause or aggravate periodontal inflammation by improving soluble UA levels. Therefore, these two above diseases may interplay with each other and even promote each other. In summary, periodontitis could aggravate tissue inflammation and destruction by increasing the level of soluble UA, and the elevated soluble UA levels in hyperuricemia or gout patients may have a deteriorating effect on the tissue destruction of periodontitis.

### Indirect effect of periodontitis on hyperuricemia

Existing cross-sectional studies, in which the prevalence of chronic kidney disease is significantly increased in patients with periodontitis, have shown that periodontitis is a risk factor for chronic kidney disease (96). Chronic kidney disease is mainly characterized by the decrease in glomerular filtration rate caused by glomerular lesions. In the later stage, it can involve renal tubules and cause secretion and reabsorption disorders. All these lesions lead to a decrease in the excretion of UA, and the kidney is responsible for 70% of the total excretion of UA. Therefore, periodontitis could lead to secondary hyperuricemia or aggravate primary hyperuricemia by increasing the prevalence of chronic kidney disease. Indeed, such a link may also be present in diabetes (9, 96).

### The results of experimental and epidemiological investigation lack sufficient theoretical support

#### The antioxidation of soluble UA and MSU crystals in periodontitis

The findings of Gharbi et al. suggested the decreased capacity to control the inflammatory destruction which was caused by the upregulated oxidative stress in the pathological tissue of periodontitis patients (97). This observation implied that periodontitis could be initiated when the balance between the production of ROS and the stress total antioxidant capacity (TAOC) was broken (97, 98). The discovery from Sakanaka et al. showed that soluble UA was related to lower periodontal inflamed surface area with statistical significance (99), supporting that soluble UA played an antioxidant role against oxidative stress and confined the inflammatory damage. Additionally, Narendra et al. supported the viewpoint that soluble UA could act as a specific antioxidant in the gingival crevicular fluid in patients with chronic or aggressive periodontitis (100, 101). The existing research is prone to the close association between periodontitis and decreased amount or activity of these antioxidants, supporting the protective effect of soluble UA in periodontitis. Accordingly, an imbalance between the production of free radicals and local antioxidants in periodontal tissue is regarded as the major pathologic pattern in periodontitis, leading to periodontal tissue destruction. El et al. proposed that soluble UA played the role of antioxidants, which could scavenge ROS and peroxynitrite in the normal human cytosol specifically in the liver, vascular endothelial cells, and human nasal secretions (74). The increased ROS in inflammatory tissue can be eliminated undoubtedly by soluble UA, resulting in the alleviation of inflammation and tissue destruction, which supports the outcome of Byun and his colleagues. Barnes et al. found a significantly decreased soluble UA level in the tissues of the periodontitis site (102). It can be inferred that soluble UA has an antioxidant effect by removing ROS produced in periodontal tissue with severely inflammatory damage, on account of the reduced level of soluble UA in the inflammation site. Novakovic et al. observed a significant positive correlation between TAOC and soluble UA in chronic periodontitis patients (103). Once again, the aforementioned research implies the protective effect of soluble UA on bone metabolism.

#### The effect of UA on the alveolar bone

On the premise of determining that hyperuricemia promotes the destruction of alveolar bone in mice, research by Sato et al. has demonstrated that obesity increases the production of soluble UA mediated by gut dysbiosis, contributing to accelerating resorption of alveolar bone in periodontitis, which processes could be blocked by allopurinol, a xanthine oxidase inhibition (104). Miricescu et al. detected a significant positive correlation between salivary levels of matrix metalloproteinase -8(MMP-8) and soluble UA in the chronic periodontitis group, as well as the positive correlation between C-terminal telopeptide of type I collagen (CTX I) and soluble UA (105). MMP-8 and CTX-I are vital bone-loss biomarkers (105), indicating soluble UA is a risk factor for periodontitis. To some extent, there is a contradiction between the protective effect of soluble UA as an antioxidant and its positive relation with MMP-8 et al. that leads to damage to periodontal tissue. Although distinct points of view about the relationship between periodontal health and antioxidants have been presented, the minority view is still for the protective and antioxidative role of soluble UA in periodontitis. To date, this issue remains incompletely investigated and scientifically controversial.

#### Epidemiological researches concerning the effect of hyperuricemia on periodontitis

Byun et al. using a cross-sectional and prospective cohort study design illustrated that hyperuricemia was associated with periodontitis, which proposed that the elevated soluble UA levels might have a protective effect on periodontitis (106). Notably, many confounding factors like medical treatment, drug intake, and dental plaque existence were not taken into consideration, which could reduce the credibility of the experiment. There could be some information bias affecting the reliability of the experimental system. On the contrary, Banu et al. found elevated levels of soluble UA in periodontitis patients compared with healthy controls (86), against the protective effect of soluble UA probably. Babaei et al. found that non-surgical periodontal treatment lowered soluble UA in periodontitis patients (107), which seemed to disagree with the aforementioned epidemiological study. Clinically, the non-surgical periodontal treatment removes most of the dental plaque that destroys periodontal tissue in pathogenesis. The reduction of soluble UA in periodontitis patients could be attributed to less cell injury and tissue damage after treatment, or soluble UA could act as the parameter of inflammation associated with it positively. These observations were opposite to the change caused by antioxidants which were in line with the former epidemiological experiment.

## Summary and outlook

Based on the foregoing, we have made some bold assumptions in this review. On account of the existing research articles on hyperuricemia and periodontitis, we have some hypotheses about the relationship between the two diseases, which may provide some ideas for future research. Periodontitis is an inflammatory disease caused by periodontal pathogens infection, while hyperuricemia is a metabolic disease caused by excessive soluble UA in the blood leading to aseptic inflammation, both of which can act on TLR to initiate the NF-κB signaling pathway. Due to the interaction between distinct signaling pathways, such as the PI3K/AKT pathway, downstream of which could initiate the NF-κB signaling pathway as well. Soluble UA can also activate the Akt-PRAS40 pathway, bringing about the inhibition of autophagy. Both LPS and MSU crystals can activate the MAPK pathway to induce inflammation, and there may be an amplification of the inflammatory magnitude. In addition, hyperuricemia may increase the number of pathogenic bacteria and then cause more serious periodontal tissue destruction. However, the effects of UA on the growth of periodontal pathogens may be specific. Not all periodontal pathogens can be promoted by soluble UA, and the suitable pH for the growth of different periodontal pathogens also has certain effects. This could explain the opposite trend of soluble UA levels in tissues from periodontitis sites compared to healthy controls in different studies.

There is no definite evidence for the effect of hyperuricemia on bone metabolism currently, but most opinions are biased toward soluble UA as a protective factor for bone metabolism. Relatively, there are still plentiful studies suggesting that soluble UA is a risk factor for bone loss under the premise of hyperuricemia, while periodontal inflammation involves alveolar bone loss. However, in the above studies, the scholars did not indicate the specific concentration range of UA that can promote or inhibit bone metabolism. Therefore, we cannot be certain that the opposite experimental results are not caused by concentration differences.

UA levels in saliva and blood of periodontitis patients were higher than those of healthy people, while another study found that UA levels in local tissues of periodontitis decreased. This discrepancy is most likely due to the differences in the periodontal pathogens. Variable control of the dominant bacteria is necessary to further determine the relationship between local and systemic UA levels in periodontitis patients and the differences from healthy controls.

The increasing prevalence of hyperuricemia and periodontitis has caused scholars in related fields to urgently explore methods of disease control and prevention from a holistic perspective. Based on existing studies, this article proposes a hypothesis that periodontitis is associated with hyperuricemia and discusses the two antithetical views coupled with listing the relevant experimental evidence. The revelation of the relationship between the two diseases will undoubtedly promote the development of periodontal medicine. As a chronic oral inflammatory disease closely related to systemic diseases, the establishment of the relationship between periodontitis and metabolic diseases will reveal the relationship between periodontitis and metabolic diseases, and promote the development of treatment and prevention of periodontitis and hyperuricemia. Currently, the number of epidemiological studies on the direct relationship between hyperuricemia and periodontitis is insufficient. Lacking assistance from professional dentists, deficiency of *in-vivo* experiments, and a tiny quantity of basic experimental research, all above limit the understanding of the relationship between hyperuricemia and periodontitis. The research on the relationship between these two diseases can be considered as a new research direction and the prospects of which will be promising in the future. In summary, to investigate and clarify the relationship between hyperuricemia and periodontitis, further research is required to determine exactly how they interact and the molecular mechanism.

## Author contributions

Literature review and manuscript drafting: WH; Manuscript revise: XL and JL; Verification of manuscript: XX, HL, and YL. All authors contributed to the article and approved the submitted version.

## Acknowledgments

The authors are very thankful for financial support by the Shandong Provincial Natural Science Foundation Youth Project (ZR2021QH251), Clinical Medicine +X Research Project of Affiliated Hospital of Qingdao University (QDFY+X2021055).

## Conflict of interest

The authors declare that the research was conducted in the absence of any commercial or financial relationships that could be construed as a potential conflict of interest.

## Publisher’s note

All claims expressed in this article are solely those of the authors and do not necessarily represent those of their affiliated organizations, or those of the publisher, the editors and the reviewers. Any product that may be evaluated in this article, or claim that may be made by its manufacturer, is not guaranteed or endorsed by the publisher.
